# An Evaluation of the Effects of Delayed Parasitism on Daily and Lifetime Fecundity of *Aphidius ervi* Haliday

**DOI:** 10.3390/insects16010003

**Published:** 2024-12-24

**Authors:** Vincenzo Trotta, Paolo Fanti, Roberto Rosamilia, Donatella Battaglia

**Affiliations:** Dipartimento di Scienze Agrarie, Forestali, Alimentari ed Ambientali (DAFE), Università degli Studi della Basilicata, Via dell’Ateneo Lucano 10, 85100 Potenza, Italy; paolo.fanti@unibas.it (P.F.); roberto.rosamilia@studenti.unibas.it (R.R.)

**Keywords:** aphids, population dynamics, ecological behaviour, fitness, parasitization rate, superparasitization, fecundity, body size

## Abstract

The study of parasitoid reproductive behaviour is crucial to understanding how parasitoids influence host population dynamics, and the strategies used by parasitoids to maximize their reproductive success. Studying how the parasitoid optimizes its reproductive resources is important as it provides information to improve the efficiency of a biological control programme. Many studies have been carried out on *Aphidius ervi* to assess the foraging behaviour of the parasitoid, but how the age of the parasitoid affects its foraging behaviour is still poorly understood. In the present study, the influence of the time elapsed between adult parasitoid emergence and the first oviposition in *A. ervi* was evaluated. Correlations between the parasitoid size and other fitness components were also investigated. Our results show how the parasitoid senescence is associated with the loss of reproductive capacity, as the late field release of parasitoids reduces their total parasitism. We also observed a parasitization rhythm independent of the parasitoid age. Possible explanations for the observed phenomena are discussed.

## Simple Summary

Predator–prey population dynamics is one of the most studied problems in population biology. Parasitoids’ reproductive behaviour is crucial in shaping host population dynamics and plays a key role in determining their reproductive strategies. Along with many biotic and abiotic parameters, the age of the adult parasitoids plays an important role in their performance. This research focuses on the variation in some biological parameters related to the fecundity of the parasitoid *A. ervi*, as a function of female age at the first oviposition. The results of the present study provide key elements on the effects of the time elapsed between parasitoid emergence and first oviposition, making it possible to predict the behaviour and performance of the parasitoid following its release in the field.

## 1. Introduction

The dynamics of the interactions between a predator and population resource is one of the most studied topics in population biology [1]. The consumption rate of a predator is limited, because even when prey is abundant and search time is practically zero, a predator still spends time handling a prey. In the Aphidiinae (a subfamily of hymenopteran parasitoids very important in biological control programmes), the rate of parasitization often decreases with increasing prey density; however, the parasitoid age can influence some aspects of its reproductive behaviour [2,3]. The study of parasitoid reproductive behaviour is then important because it is key to understanding how parasitoids influence host population dynamics and interact with their lifecycle, and to determining the strategies used by the parasitoid to maximize its reproductive success.

A substantial body of research has been conducted on the parasitoid *Aphidius ervi* Haliday (Hymenoptera: Braconidae), an aphid parasitoid widely used as a model species in behavioural, physiological, and molecular studies [4,5,6,7,8,9]. Like other koinobiont parasitoids, *A. ervi* can oviposit in any aphid instar as it is able to regulate aphid physiology, behaviour, and reproduction to meet the nutritional and physiological requirements of the parasitoid larvae [5,10]. The study of parasitoid reproductive behaviour is essential to understand and determine the evolutionary history and behaviour of the host choice, fecundity, and sex ratio [11]. The success of biological control is contingent upon a comprehensive understanding of the organisms involved, encompassing both pest and beneficial species, as well as their intricate interactions.

*Aphidius ervi* can be considered a pro-synovigenic species; i.e., females emerge with a certain number of mature eggs in their body, but continue to produce and mature others during their lifetime [12,13]. The number of eggs a female parasitoid lays in her lifetime is determined by the interaction of three processes: the number of hosts she encounters, the number of eggs she is able to mature in her lifetime, and the handling time before she lays them.

During the same time interval, individual differences in the percentage of eggs laid may be determined by differences in the number of ovarioles (assuming that the pattern of ovogenesis in the ovarioles is fairly constant [14]), by temporal differences in the maturation of the eggs themselves, or by both of these factors. The variability in the number of aphids attacked by a parasitoid may be contingent upon the age of the females [15,16]. In addition, the phenomenon of superparasitism (a host being attacked more than once by a female) can reduce the efficacy of parasitoids [17]. The phenomenon of superparasitism has been observed in *A. ervi*, which is only capable of recognizing already parasitized hosts a minimum of six hours after the parasitization event [18].

Knowledge of the parasitoid’s emergence, maturation, and oviposition during the day can be used to understand its ecology and the evolution of its reproductive strategy, and is useful for the development and improvement of a biological control programme. The reproductive rate of a parasitoid is also influenced by its body size. Body size constitutes a specific and significant life history trait that influences the metabolic rate, which in turn affects an organism’s demand for energy and its reproductive success [19]. Other life history traits such as the energy demand, reproductive success, and sex ratio are directly influenced by body size [11,20,21]. It is therefore evident that body size has a significant effect on the fitness of an organism. The insect wing, with its network of veins, is regarded as an excellent model system for investigating body size [5,22,23,24].

Together with many biotic and abiotic parameters, parasitoid age plays an important role in their performance [15,16,25]. There is a notable lack of research on the reproductive behaviour of *A. ervi*, which presents a significant obstacle to understanding and determining the evolutionary history and behaviour of the host choice, fecundity, and sex ratio. The majority of studies have not analyzed other factors that may influence the functional response of parasitoids, such as fecundity, age, and superparasitization. This study investigated the reproductive behaviour of *A. ervi* in a trophic model system consisting of the parasitoid and its host aphid *Acyrthosiphon pisum* (Harris), as this is a key element in implementing and improving a successful biological control strategy. The present study evaluated a number of biological parameters related to the reproductive behaviour of the parasitoid of *A. ervi*, namely longevity, fecundity, superparasitization, variation in parasitization throughout life and throughout the day, and correlations between parasitoid size and its reproductive behaviour as a function of female age at the first oviposition.

## 2. Materials and Methods

### 2.1. Insects and Experimental Procedure

*Aphidius ervi* parasitoids were obtained from Koppert Italia (Bussolengo, Italy) and reared in a laboratory on the pea aphid *Acyrthosiphon pisum*. The aphids were collected from *Medicago sativa* near Salerno, Italy, and reared in the laboratory on broad bean plants (*Vicia faba* L., cv. aguadulce). Aphid and parasitoid cultures were maintained in two separate environmental chambers at 20 ± 1 °C and 75 ± 5% relative humidity (RH), under an 18:6 h light/dark (L/D) cycle.

To obtain synchronized aphids, 30 adult apterous females were placed on a broad bean plant in plastic containers sealed with tulle and removed after 24 h. The first instar aphids on the plants were counted, leaving approximately 100 aphids on each plant. As small aphids can escape counting, after 24 h, the aphids, which had become second instar, were counted again, leaving 100 aphids on each plant. After 24 h, i.e., 72 h after hatching, the nymphs were counted again, always leaving 100 individuals (which had become third instars) on each plant. These aphids were used for the parasitization experiments because aphids at the beginning of the third nymphal instar allow high parasitization success [5]. Under our experimental conditions, 100 aphids are more than the maximum number of aphids that a single *A. ervi* female can parasitize in 24 h.

To obtain parasitoids of known age, mummies were removed daily from the mass rearing container and individually placed in glass tubes sealed with cotton and containing a drop of honey. The tubes with the mummies were inspected several times during the day, until the emergence of adult parasitoids. Once adults emerged, the time and day of emergence were recorded, sex was determined, and females were allowed to mate and left with a male for half an hour. After mating, the females were separated from the male. An aphid was then placed inside the tube, and after several stings with the ovipositor, the aphid was removed from the tube.

Newly hatched 1 h old female parasitoids, 24 h old female parasitoids, 48 h old female parasitoids, and 72 h old female parasitoids were used for the tests. At each age (1, 24, 48, and 72 h), each female was provided with her first opportunity for oviposition. To ensure the survival of the females up to 72 h and to minimize stress, the females were transferred to larger tubes after 24 h, and after 48 h, they were placed in small plastic beakers with some honey and commercial candied orange peel, and the container was sealed with gauze. When the parasitoids had reached the age required for testing, the females were used individually for parasitization.

### 2.2. Parasitization Rate and Longevity

To analyze how the parasitization rate of *A. ervi* changes over time, daily parasitization was analyzed throughout the entire life of the parasitoid, thus estimating the longevity of individual parasitoids in addition to their overall fecundity and parasitization rate over time. Experiments were performed using females that had been held for 1, 24, 48, or 72 h before being offered hosts for oviposition, in a climatic chamber, at a temperature of 20 °C, and 75% R.H., under long day conditions: 16/8 L/D.

For each parasitization test, a single plant with 100 third nymphal instar aphids was placed inside a Plexiglas cylinder (height: 35 cm, diameter: 25 cm). Together with the infested plant, the tube containing the female parasitoid of known age was placed in the cylinder with a small strip of honey on a piece of paper. The cylinder was then sealed at the top with tulle to ensure good ventilation and was placed in a climatic chamber for 24 h. After 24 h, the parasitoid was removed from the experimental arena, the plant was transferred to a plastic container sealed with tulle, and each aphid was carefully transferred together with the plant, also collecting aphids that had fallen from the plant using a soft brush. The parasitoid was returned to the cylinder, a new plant with aphids on it was placed inside, and the parasitoid was returned to the climatic chamber for 24 h; this process was repeated until the parasitoid died naturally. Plastic containers with the plants infested with parasitized aphids were placed in the climatic chamber, at 20 °C and 75% R.H. Wing area was measured to assess the size of the female parasitoid following her death [4,5,20,21,23,24]. Longevity was estimated as the number of days a female was used for the test plus the number of days she spent in the tube before the test. For each of the four female age groups (1, 24, 48, and 72 h), two independent replicates of six (five in some cases) individuals were generated.

Three days after parasitization, aphids were collected from the plant, counted, frozen, dissected, and examined under a stereomicroscope to detect the presence of parasitoid larvae within the aphids. For endoparasitoids like *A. ervi*, there is no way of knowing for sure if an aphid hosts a parasitoid egg or larva without dissecting it. For this reason, all aphids from each trial were dissected and the presence and number of larvae found within the host were recorded. The parasitization rate per day was estimated as the number of aphids hosting at least a parasitoid larva. Superparasitization was estimated as the number of aphids with two parasitoid larvae. Parasitoid fecundity was calculated as the total number of aphids parasitized by a female during her lifetime, including superparasitism events. Fecundity was also analyzed using the cumulative parasitization per female over time for each experimental group, as the use of cumulative offspring production allows the visualization of the maximum offspring production and the time required to reach it.

Approximately 20,000 aphids were dissected for this experiment.

### 2.3. Parasitoid Body Size

**Figure 1:**
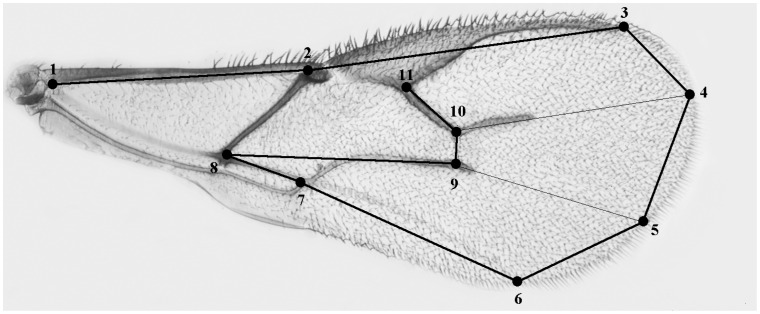
Wing of *A. ervi*. Landmarks used to calculate and analyze wing size (1 to 11).

The wing size of the females used for the tests was measured. Wing sizes of the parasitoids from each experimental group were measured as centroid size [5,24]. Wing size is positively correlated with body size as a whole and is a considerably easier feature to measure accurately [24]. It has also been shown that in *A. ervi*, wing CS was highly correlated with thorax length, head width, and tibia length [5]. Briefly, left wings were dissected, dehydrated in ethanol, and mounted on glasses in lactic acid/ethanol (6:5). Wing images were captured using a Nikon optical microscope mounting a digital camera (Nikon, Tokyo, Japan). The 50 × optical magnification was subsequently enlarged through a 2 × digital zoom. The images were used to record 11 morphological landmarks (Figure 1) and wing size was estimated as centroid size (CS). CS is defined as the square root of the sum of the squared distances of a set of landmarks from their geometric centre (the centroid).

### 2.4. Variation in the Parasitization Rate Throughout the Day

In order to assess how the parasitization of *A. ervi* varies throughout the day, tests were carried out in the climatic chamber at a temperature of 20 °C, RH of 75%, and a photoperiod of 16/8 L/D, according to the following protocol. The mated females were placed in Plexiglas cylinders, also used for the other tests, inside which was a plant infested with 50 aphids at the third nymphal stage. The 24 h period was divided into four fractions, three light periods of 4 h each and one 12 h period consisting of 4 h of light and 8 h of darkness (hours 08–12, 12–16, 16–20, and 20–08). At the end of each time fraction, the female was removed and transferred to a new cylinder containing a new infested plant. After three days, the aphids were collected and dissected to assess the presence of parasitoid larvae and thus the parasitization value in the fraction of the day considered. Two independent replicates of nine and eight individuals were generated. Only females of 24 h were used for these experiments.

### 2.5. Statistical Analysis

Data on parasitoid longevity, total fecundity, superparasitization, wing size, and parasitoid fecundity during the day were analyzed using linear models (LMs) as the homoscedasticity and normality assumptions for these ANOVAs were checked and met in the data. The following LM was applied:

Y = µ + Female Age + Replicate {Female Age} + ε

where Y is the dependent variable with a normal distribution, “Female Age” (four levels: 1 h, 24 h, 48 h, and 72 h old parasitoid females) is the main factor, and “Replicate” (two levels) is nested within “Female Age”. When the effect of “Female age” was significant, a Tukey *post hoc* test for multiple comparisons of means was also performed to detect significant differences between experimental groups. The same LM was also applied to the data on parasitoid fecundity during the day, but in this case the main factor was “Day interval” (four levels: hours 08–12, 12–16, 16–20, and 20–08) and “Replicate” nested within “Day Interval”.

For the analysis of fecundity over time, the cumulative number of parasitized aphids per female was used. In addition, the use of cumulative parasitization values makes it possible to visualize the maximum parasitization values per female age and the time required to reach them. The best fit for the cumulative productivity over time was given by second-order polynomials (R^2^ > 0.99 in all the cases). As the data were normally distributed, the mean cumulative parasitization values per female were then analyzed by ANCOVA with “time” as a covariate, as this is a continuous independent variable that influences the dependent variable but is not of primary interest to the study. The results of the ANCOVA make it possible to identify differences in the slopes of the polynomials indicating differences in “Female age” over time.

All insect data were analyzed using the statistical software R version “4.4.1” [26].

## 3. Results

### 3.1. Parasitoid Longevity, Total Fecundity, and Superparasitization

**Figure 2:**
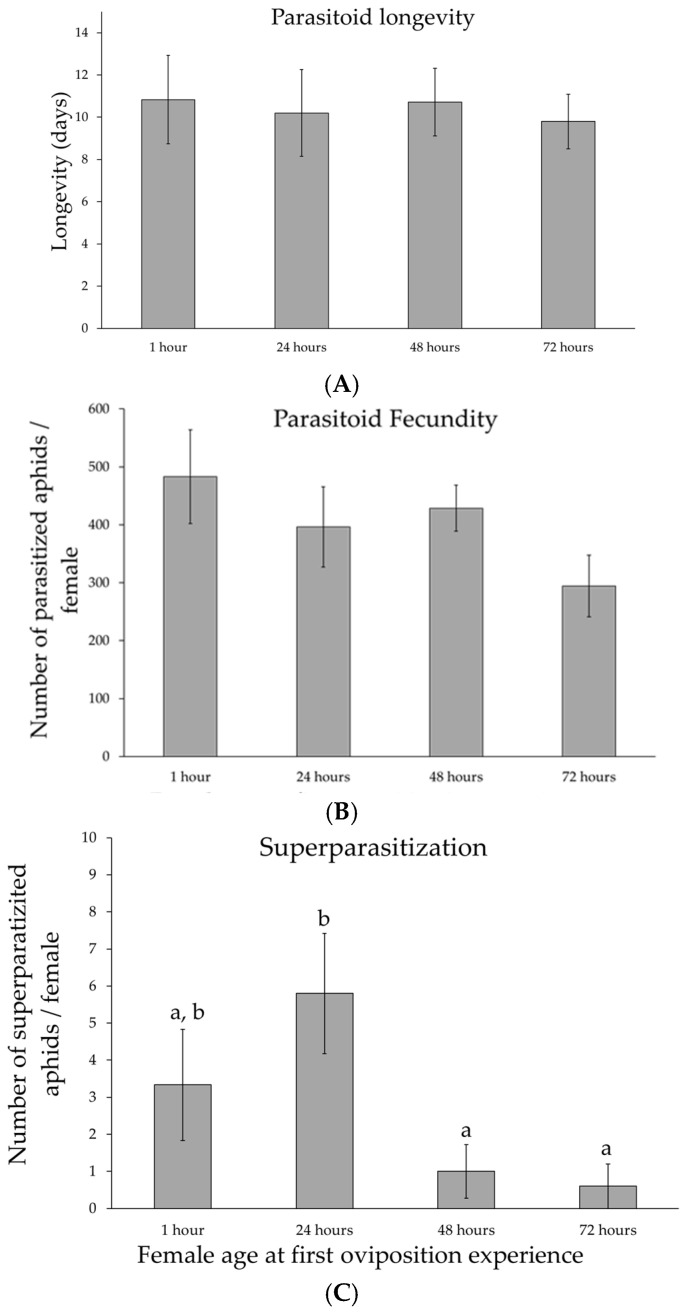
Reproductive behaviour of the parasitoid of *Aphidius ervi*. Mean values (±standard errors) of the parasitoid longevity (**A**), fecundity (**B**), and superparasitization (**C**) as a function of female age at the first oviposition—1 h: N = 12; 24 h: N = 11; 48 h: N = 11; 72 h: N = 11. Different lower case letters indicate Tukey *post hoc* differences.

Parasitoid longevity was determined by measuring the lifespan of the females used in the experiment (Figure 2A). Female ages refer to the time an experimental female spends in a tube before being used for parasitization. Female age at first oviposition did not significantly affect parasitoid longevity (F_3,37_ = 0.06, *p* = 0.98). No significant difference was found between replicates (F_4,37_ = 0.36, *p* = 0.98).

As for longevity, parasitoid total fecundity (Figure 2B) showed no significant differences among female age at the first oviposition (F_3,37_ = 1.4, *p* = 0.28) and between replicates (F_4,37_ = 0.51, *p* = 0.73).

Regardless of female age at first oviposition, superparasitization events occur early in life, during the first three days. Mean values of superparasitization (aphids with two parasitoid larvae inside them) are shown in Figure 2C. Female age significantly affected superparasitization (F_3,37_ = 4.23, *p* = 0.024). No significant difference was found between replicates (F_4,37_ = 1.52, *p* = 0.25). Parasitoid females used immediately for parasitization experiments (1 h) or after 24 h showed higher levels of superparasitization compared to 48 h and 72 h old females (Tukey multiple comparisons of means: *p* < 0.05 in both cases).

### 3.2. Cumlative Fecundity over Time

**Figure 3:**
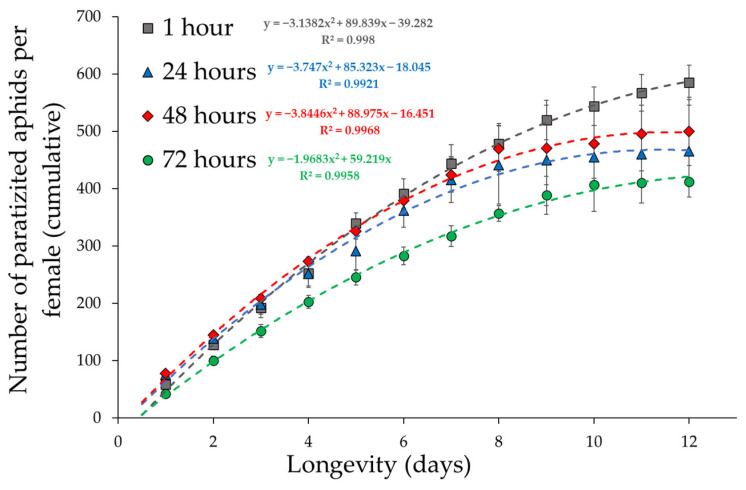
Cumulative fecundity per female over time (±standard error) of the four female ages at the first parasitization. Few females survived after the twelfth day, so oviposition values after this day were excluded from the analyses.

The cumulative number of parasitized aphids over time was used as an indicator of the fitness of the experimental groups to better understand the reproductive behaviour of the parasitoid. The best fit for cumulative fecundity over time was given by second-order polynomials (Figure 3).

An ANCOVA was then performed to identify differences in the slopes of the polynomials, indicating differences in reproductive rates between female ages at first oviposition. The interaction between the polynomial regressions and “Female age” was significant (F_6,36_ = 31.7, *p* < 0.001), indicating that female fecundity over time varied greatly with age at first oviposition. Parasitoid females that spent three days in the vials before the first oviposition had a lower performance over time compared to the other experimental groups.

### 3.3. Correlation Between Wing Size and Reproductive Traits

As expected, although there were small size differences among females, no significant differences in wing size were found for “Female age” at first oviposition (F_3,37_ = 0.6, *p* = 0.62), as parasitoids were randomly assigned to the different experimental treatments. We then investigated the correlation between parasitoid size, longevity, total fecundity, and superparasitization. No statistically significant correlations were found between body size and the reproductive traits of *A. ervi* as a function of the different female ages at the first oviposition (Figure S1).

### 3.4. The Parasitization Rate During the Day

Differences in the parasitization rate during the 24 h cycle were found for 24 h old females (F_3,60_ = 292, *p* < 0.001), but not between replicates within the time interval (F_4,60_ = 0.14, *p* = 0.96).

**Figure 4:**
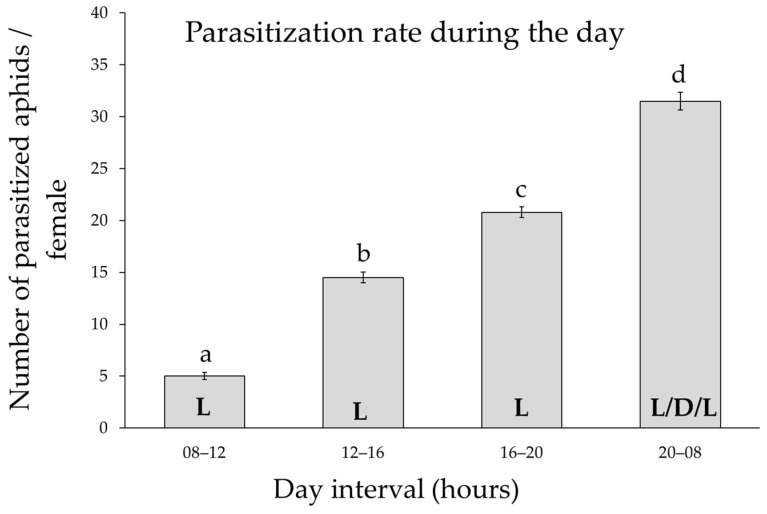
The parasitization rate during the 24 h cycle. Mean values (±standard errors) of the parasitoid rate of 24 h old females as a function of four different time intervals during the day. N = 17. L: light; D: dark. Different lower case letters indicate Tukey *post hoc* differences.

The rate of parasitization was very low in the morning, then increased during the afternoon/evening and peaked between 20:00 and 08:00 a.m. (Figure 4). During this last interval, it was observed that the highest activity of the females occurred in the early morning hours with light.

## 4. Discussion

This work was carried out to analyze how the oviposition of the hymenopteran parasitoid *Aphidius ervi* varies over its lifetime and whether it is influenced by female age at first oviposition. Parasitoids are organisms that reproduce both sexually and asexually within the same lifecycle, often facing a significant level of inbreeding in natural populations.

The analysis of the longevity data shows that the average lifespan of the parasitoids was about 10 days, irrespective of the time spent in the tube before oviposition; i.e., it is the same for females of the different age classes considered. This result is consistent with data reported in the literature [27,28,29], and suggests that there is no trade-off between longevity and reproduction in this parasitoid. There are many possible explanations for this result, but it is interesting to note that in nature, mortality is strongly influenced by different environmental factors (temperature, host finding, etc.) and in the present experiment, the females were all kept in the same optimal environment.

The total fertility data show no significant differences between the different ages of females at first oviposition. The differences between the groups of females’ age at first parasitization are particularly evident when the cumulative curves are used to analyze productivity. When considering parasitism data over time, it is evident that 72 h old females had lower productivity values than females in the other groups, and this trend was statistically significant. Since all parasitoid females have their own load of mature eggs at the emergence (varying between 60 and 80 eggs), the 72 h old females should also have this reproductive potential, so the reduction in parasitization could be due to the reduction in the number of mature eggs at the time of the test. The reduction in the number of eggs can be due to either the reabsorption or expulsion of mature eggs and subsequent inhibition of the maturation of new eggs. Both possibilities (reabsorption or expulsion) represent an energy cost to the parasitoid, with the energy expended in saving eggs resulting in a reduction in the number of eggs that the parasitoid can mature in the remainder of its life. It is therefore possible that after 3 or more days of inactivity, physiological mechanisms come into play that inhibit (even partially) the production and/or maturation of new eggs throughout the lifespan. New and different experiments are needed to confirm or reject this possible explanation. From a behavioural point of view, 72 h old females are in a highly stressful situation because they have been kept for three days in an environment that is very different from their natural environment. When they return to an environment similar to the natural environment, they show a reduced ability to find hosts, which is reflected in lower productivity.

When an insect finally exhausts its egg load, its reproductive period ends. An important cost of senescence is the loss of reproductive capacity. These events occur simultaneously with a reduction in host exploitation as a result of the limited number of eggs available, or with the production of low-quality offspring shortly before the end of the egg load [11].

When considering superparasitization, the differences between females of different ages at the first parasitization were significant. In particular, superparasitization was higher in 1 h old and 24 h old females than in the other two groups. Superparasitization was also concentrated in the first few days of the experiment, when the percentage of parasitized aphids is highest. Because females have a greater supply of eggs, they have a greater chance of stinging aphids that have already been parasitized. The female parasitoid is able to recognize hosts already parasitized by the presence of a marker pheromone [30], but if the number of unparasitized hosts is low, cases of superparasitization can still occur.

Fitness traits are strongly influenced by body size [11,20,21], but in the present study, no significant differences in body size were found between females of different age classes. The choice of the present experimental design probably led to the lack of significant correlations between productivity, longevity, and size observed by many authors in insects [5,11,20,21].

The results on the parasitization rate during the day are consistent with the bibliography [31,32,33,34] and showed that egg laying by the parasitoid varies throughout the day. During the dark period, *A. ervi* females oviposit and sting few aphids, although they have a sufficient supply of eggs [35]. This suggests that the oviposition pattern of *A. ervi* is determined by exogenous light-related factors, as this parasitoid is a diurnal insect. Light therefore influences the movement, oviposition, and emergence rhythms of *A. ervi* [13]. The emergence of adults during the light phase probably coincides with conditions that increase the chances of locating the host habitat, finding the host and the opposite sex for mating. Since most of the parasitization activity is concentrated very early in the morning and in the second phase of the day, it could be assumed that during the first phase of the day, the parasitoid activity is focused on exploring the environment, searching for the host, while after this period, it concentrates on parasitizing. Another possible explanation for the difference in parasitization during the day could be related to the circadian rhythms of the parasitoid, which are characteristic of the species studied [33,34]. Our results suggest that parasitoid field releases should be concentrated in the evening, to maximize parasitization by *A. ervi*. Morning or afternoon releases could lead to a reduction in parasitization, as the parasitoid might spend time exploring the environment instead of parasitizing.

## 5. Conclusions

Our results suggest that a late release of parasitoids reduces their proportion of total parasitism, and that the number of individuals to be released in the field should be determined on the basis of parasitoid age. We have also shown that there is a circadian oviposition rhythm independent of parasitoid age, which could be useful for planning biocontrol actions. Our findings indicate that there is no significant variation in the reproductive behaviour of the parasitoid among females reared under identical standard conditions. When using parasitoids in biological control programmes, knowledge of the time elapsed between reaching the reproductive age and use in the field allows the behaviour of the parasitoid on arrival from the biofactories to be determined. Knowing the age of parasitoids prior to field release can be useful in determining the best density for field release of these valuable insects.

## Data Availability

The raw data supporting the conclusions of this article will be made available by the authors upon request.

## Supplementary Materials

The following supporting information can be downloaded at: www.mdpi.com/article/10.3390/insects16010003/s1, Figure S1: Scatter plots of body size and reproductive traits of Scatter plots of body size and reproductive traits of *A. ervi*.
