# Supplementary material for: An Evaluation of the Effects of Delayed Parasitism on Daily and Lifetime Fecundity of Aphidius ervi Haliday
Source: Insects. 2024 Dec 24;16(1):3. doi: 10.3390/insects16010003 (PMC11766066; doi:10.3390/insects16010003)
Supplement: Supplementary file 1 [file insects-16-00003-s001.zip › insects-3374060-supplementary.pdf]

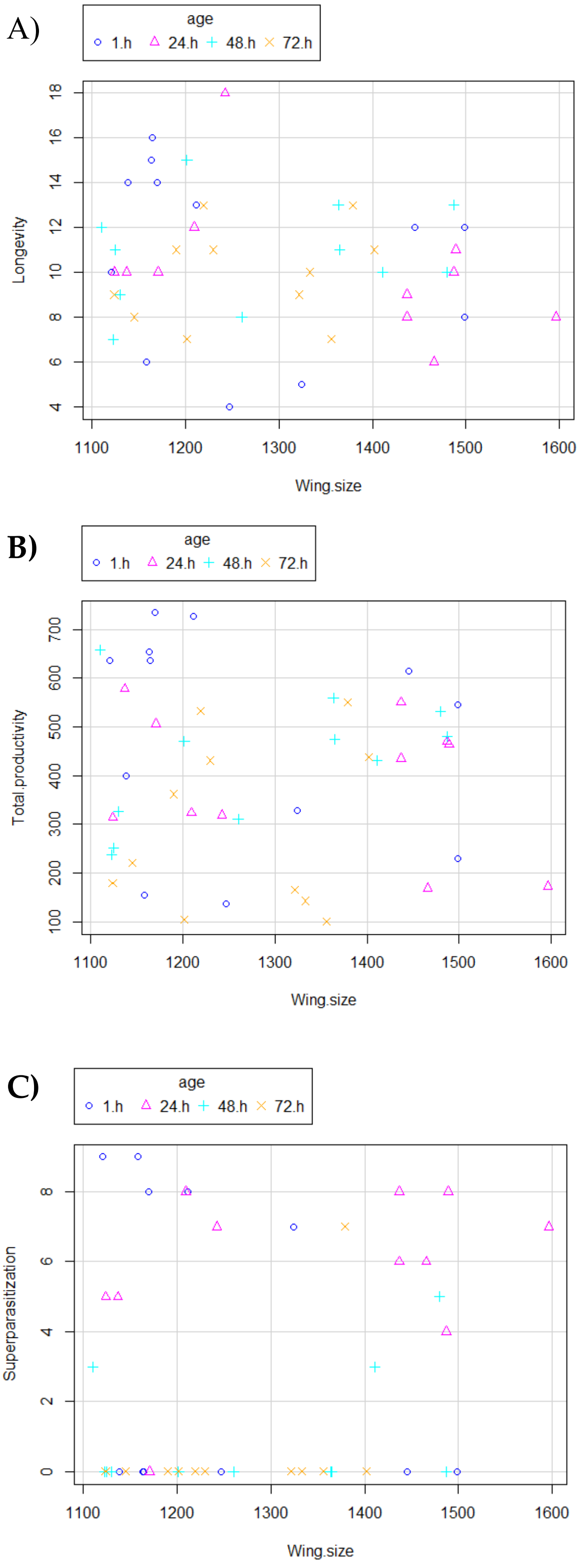

Figure S1. Scatter plots showing the relation between parasitoid size and A) longevity, B) total fecundity and C) superparasitization. No statistically significant correlations were found between body size and the reproductive traits of *A. ervi* as a function of the different female ages at the first oviposition.
